# Association between Shift Work and Health Outcomes in the General Population in China: A Cross-Sectional Study

**DOI:** 10.3390/brainsci14020145

**Published:** 2024-01-30

**Authors:** Nan Gao, Yongbo Zheng, Yingbo Yang, Yuetong Huang, Sanwang Wang, Yimiao Gong, Na Zeng, Shuyu Ni, Shuilin Wu, Sizhen Su, Zhibo Zhang, Kai Yuan, Le Shi, Zhaohui Zhang, Wei Yan, Lin Lu, Yanping Bao

**Affiliations:** 1 The First Affiliated Hospital of Xinxiang Medical University, Xinxiang Medical University, Xinxiang 453199, China; 50210105025@stu.xxmu.edu.cn (N.G.); 50210101044@stu.xxmu.edu.cn (Y.Y.); 2 Peking University Sixth Hospital, Peking University Institute of Mental Health, NHC Key Laboratory of Mental Health (Peking University), National Clinical Research Center for Mental Disorders (Peking University Sixth Hospital), Peking University, Beijing 100191, China; yongbozheng@bjmu.edu.cn (Y.Z.); yuetonghuang@stu.pku.edu.cn (Y.H.); 2022103020021@whu.edu.cn (S.W.); 1801111460@pku.edu.cn (Y.G.); sizhensu@bjmu.edu.cn (S.S.); 2211110679@bjmu.edu.cn (Z.Z.); yuankai@pku.edu.cn (K.Y.); leshi@bjmu.edu.cn (L.S.); weiyan@bjmu.edu.cn (W.Y.); 3 Peking-Tsinghua Centre for Life Sciences and PKU-IDG/McGovern Institute for Brain Research, Peking University, Beijing 100191, China; 4 Department of Psychiatry, Renmin Hospital of Wuhan University, Wuhan 430060, China; 5 National Institute on Drug Dependence and Beijing Key Laboratory of Drug Dependence, Peking University, Beijing 100191, China; zengna19@bjmu.edu.cn (N.Z.); nishuyu@pku.edu.cn (S.N.); 2211210073@stu.pku.edu.cn (S.W.); baoyp@bjmu.edu.cn (Y.B.); 6 School of Public Health, Peking University, Beijing 100191, China; 7 Research Unit of Diagnosis and Treatment of Mood Cognitive Disorder, Chinese Academy of Medical Sciences (No. 2018RU006), Beijing 100191, China

**Keywords:** shift work, health outcomes, psychiatric disorders, mental health symptoms, physical disorders

## Abstract

Shift work may adversely affect individuals’ health, thus, the current study aimed to investigate the association between shift work and health outcomes in the general population. A total of 41,061 participants were included in this online cross-sectional survey, among which 9612 (23.4%) individuals engaged in shift work and 31,449 (76.6%) individuals engaged in non-shift work. Multiple logistic regression analyses were conducted to explore the association between shift work and health outcomes (psychiatric disorders, mental health symptoms, and physical disorders). In addition, associations between the duration (≤1 year, 1–3 years, 3–5 years, 5–10 years, ≥10 years) and frequency of shift work (<1 or ≥1 night/week) and health outcomes were also explored. The results showed that compared to non-shift workers, shift workers had a higher likelihood of any psychiatric disorders (odds ratios [OR] = 1.80, 95% CI = 1.56–2.09, *p* < 0.001), mental health symptoms (OR = 1.76, 95% CI = 1.68–1.85, *p* < 0.001), and physical disorders (OR = 1.48, 95% CI = 1.39–1.57, *p* < 0.001). In addition, inverted U-shaped associations were observed between the duration of shift work and health outcomes. These results indicated that shift work was closely related to potential links with poor health outcomes. The findings highlighted the importance of paying attention to the health conditions of shift workers and the necessity of implementing comprehensive protective measures for shift workers to reduce the impact of shift work.

## 1. Introduction

With the development of the social economy, the working hours of some industries and factories have been extended to nighttime. Shift work usually involves work schedules inconsistent with customary daytime work, including regular morning, evening, and night work, as well as roster work and rotating three-shift work [1]. For example, in China and Korea, 36.1% of employees undertake shift work [2]. And in the United States, one-fifth of the population are shift workers or work night shifts outside of standard working hours (8:00–17:00) [3]. As time goes by, shift work has gradually been recognized as a serious occupational hazard, affecting about 15–20% of workers worldwide [4]. Recently, the impact of shift work on the long-term health and safety of staff and on social burdens has been emphasized [5].

Shift work may disturb an individual’s circadian system with alterations to sleep-activity patterns, suppression of melatonin production, and deregulation of circadian genes involved in multiple pathophysiological pathways [6,7]. As for now, shift work has been reported to increase the risk of cardiovascular diseases [5,8,9], metabolic diseases [3,10], cancer [11,12], cognitive disorders [13,14,15,16], mortality, and so on. Shift workers’ workplaces, relationships with coworkers, and individual characteristics are all factors that may influence their health conditions [17]. Some studies have found that shift work increases the risk of anxiety and depression [13,18], whereas others suggest that shift workers have better psychiatric well-being and mental health than non-shift workers [19]. In addition, many previous studies focus merely on specific occupation populations such as those from healthcare, industrial manufacturing, mining, transport, communication, leisure, and hospitality sectors, as well as on specific health outcomes such as coronary heart disease, asthma, diabetes, and breast cancer. Hence, there is a lack of study on the comprehensive health effects of shift work in the general population without limiting it to certain occupations.

Previous evidence showed that the frequency and duration of shift work had an impact on the relationship between shift work and some specific health outcomes. A study based on UK Biobank found that 3–8 nights/month, not more than 8 nights/month, of night shift work was associated with a higher risk of coronary heart disease [5]. As well, the risk of diabetes and breast cancer was shown to be associated with more frequent shift work [10,20]. In a cohort study of female nurses followed for 24 years, it was found that longer lifetime durations of night shift work were associated with decreased odds of healthy aging [21]. More research is needed on the inconsistent association between health outcomes and shift frequency [22,23].

Considering that most previous research on shift work and health outcomes focused only on specific diseases [24], this current cross-sectional study aimed to explore this relationship more comprehensively and systematically. We hypothesized that shift work would increase the risk of poor health outcomes (including psychiatric disorders, mental health symptoms, and physical disorders) in the general Chinese population. In addition, the frequency and duration of shift work would be associated with poor health outcomes in this population as well.

## 2. Methods

### 2.1. Study Design, Participants, and Data Collection

This cross-sectional study was approved by the Ethics Committee of the Sixth Hospital of Peking University (Institute of Mental Health). An online survey was conducted among the general population in 34 provinces in China from 12 June to 17 July 2022, and informed consent was obtained from all respondents. This self-designed online questionnaire was distributed on the health page of the Chinese website Joybuy; the survey link was posted on the website, and registered members received the link to the questionnaire and voluntarily filled out the questionnaire anonymously [25,26]. Convenience sampling was used to post the link to the questionnaire on the page.

### 2.2. Assessment of Shift Work

Participants who reported having a job were asked if their current job involved shift work. Shift work is a work schedule that falls outside of normal daytime working hours (9:00–17:00) or multiple shifts on a rotating basis (such as rotating between morning, afternoon, and evening). The answers included ‘never/rarely, sometimes shift work (<1 night/week), regular shift work (≥1 night/week)’.

Participants who answered ‘sometimes shift work (<1 night/week), regular shift work (≥1 night/week)’ were further asked the number of years they had been employed in shift work, with the following choices: ≤1 year, 1–3 years, 3–5 years, 5–10 years, and ≥10 years.

### 2.3. Assessment of Health Outcomes

Three types of health outcomes were assessed, including psychiatric disorders, mental health symptoms, and physical disorders. The presence of psychiatric disorders was assessed by a self-report questionnaire confirming the diagnosis by a psychiatrist of the following disorders: schizophrenia, anxiety, depression, obsessive compulsive disorder, and bipolar disorder.

Mental health symptoms, including anxiety, depression, and insomnia, were measured by the 2-item Patient Health Questionnaire (PHQ-2) [27,28], the 2-item Generalized Anxiety Disorder Scale (GAD-2) [29], and the 7-item Insomnia Severity Index (ISI) [30,31]. All measures were validated for use in Chinese [32,33,34]. Classification of the presence of poor mental health symptoms according to the total scale scores of measures was as follows: PHQ-2, normal (0–3) and abnormal (4–6) for depression [27], with a Cronbach’s alpha of 0.78 [33,35]; GAD-2, normal (0–3) and abnormal (4–6) for anxiety [36], with a Cronbach’s alpha of 0.82 [37]; and ISI, normal (0–7) and abnormal (≥28) for insomnia [30], with a Cronbach’s alpha of the Chinese version of 0.81 [34]. Scores of participants greater than the cutoff threshold indicate potential mental health symptoms.

Also, the following physical disorders were assessed with a self-reported questionnaire confirming the diagnosis by a physician of hypertension, coronary heart disease, hyperlipidemia, cerebrovascular disease or stroke, brain injury due to trauma, migraine, diabetes, chronic obstructive pulmonary disease, asthma, thyroid disease, arthritis, chronic pain, cancer, Parkinson’s disease, epilepsy, dementia, or mild cognitive impairment.

### 2.4. Covariates

Three aspects of covariates were considered, including demographic information, lifestyle, and COVID-19 status. Demographic information of the participants included age, sex, ethnicity (ethnic Han and ethnic minorities), living area (urban or rural), level of education, marital status, type of jobs, and monthly family income. Lifestyle included smoking, drinking, diet, body mass index (BMI), exercise habits, sedentary behavior, and napping habits. COVID-19 statuses include the infection status of participants and experience with quarantine during COVID-19 epidemics [16].

### 2.5. Statistical Analysis

Data analyses were performed using IBM SPSS Statistics, version 25 (IBM Corporation, Armonk, NY, USA). Descriptive statistics were used to represent demographic information, napping habits, and information related to COVID-19. The proportions of health outcomes were reported as the percentages of cases by shift work.

Participant characteristics were described using the mean (standard deviation, SD) for continuous variables and proportions for categorical variables. A *t* test and χ^2^ test were applied to examine the statistical significance of continuous variables with normal distribution and categorical variables in different shift work status groups, respectively. Multivariable logistic regression analysis was performed to explore the association between shift work and health outcomes (i.e., psychiatric disorders, mental health symptoms, and physical disorders), with all covariates mentioned above included in the model by Wald tests. Similarly, the association of frequency (<1 or ≥1 night/week) and years of shift work (≤1 year, 1–3 years, 3–5 years, 5–10 years, ≥10 years) with health outcomes were further explored using multivariable logistic regression analysis. Before using the multivariable logistic model, multicollinearity among the independent variables was assessed by calculating the variance inflation factor (VIF). A VIF greater than 5 indicates multicollinearity [38]. Separate models were performed when included independent variables were multicollinear, excluding highly correlated covariates [39]. For all logistic regression analyses, odds ratios (ORs) and 95% confidence intervals (CIs) were calculated. Two-sided Wald tests were used to determine the statistical significance of the ORs within the regression models. The level of significance was set to *p* < 0.05 for logistic regression, and Bonferroni correction was performed to adjust for multiple comparisons.

## 3. Results

### 3.1. Demographic Characteristics

A total of 42,368 participants from 34 provinces in China completed the consent and questionnaire. Data from participants with responses lasting less than 7 min, those under the age of 15, and any duplicate or impaired responses were excluded. Finally, 41,061 individuals were included in the study.

Of the 41,061 participants, 24,247 (59.1%) were female, and the mean (SD) age was 36.24 (9.51) years; 9612 (23.4%) engaged in shift work with 3821 (39.8%) being female. Shift workers reported higher rates of males (60.2% vs. 35.1%), lower education level (22.1% vs. 18.0%), manual work (22.5% vs. 16.6%), smoking (21.2% vs. 9.8%), alcohol consumption (10.5% vs. 5.2%), and low sedentary behavior (40.4% vs. 35.1%) than non-shift workers and lower rates of normal weight (50.1% vs. 56.5%), healthy diet (41.3% vs. 50.2%), regular exercise habit (23.7% vs. 25.8%), low income (27.7% vs. 30.6%), and frequent napping behavior (39.8% vs. 47.3%) compared to non-shift workers, with a *p* value < 0.001. In relation to COVID-19, a higher proportion of shift workers were infected (5.4% vs. 3.1%, *p* <0.001) and quarantined (9.4% vs. 6.7%, *p* < 0.001) than non-shift workers. Table 1 presents the demographic characteristics of the study population according to current shift status.

### 3.2. Association of Shift Work with Health Outcomes

**Figure 1:**
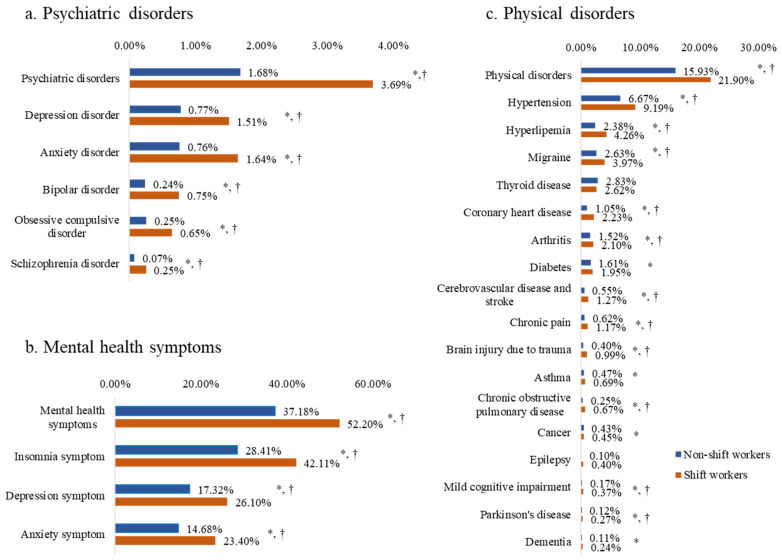
Proportions of health outcomes in shift workers and non-shift workers. * *p* < 0.05 in uncorrected tests. † *p* < 0.0018 in Bonferroni correction tests for multiple comparisons.

Figure 1 presents the proportions of each health outcome in shift workers and non-shift workers. For the entire sample, the proportion of participants practicing any psychiatric disorder, mental health symptom, and physical disorder were 2.2%, 40.7%, and 17.3%, respectively. Participants in the shift work group reported higher proportions of all three health outcomes when compared to the non-shift workers: psychiatric disorders (3.7% vs. 1.7%, *p* < 0.001), mental health symptoms (52.2% vs. 37.2%, *p* < 0.001), and physical disorders (21.9% vs. 15.9%, *p* < 0.001). The results for the number of detections and rates for health outcomes are shown in Table S1 in the Supplement.

**Figure 2:**
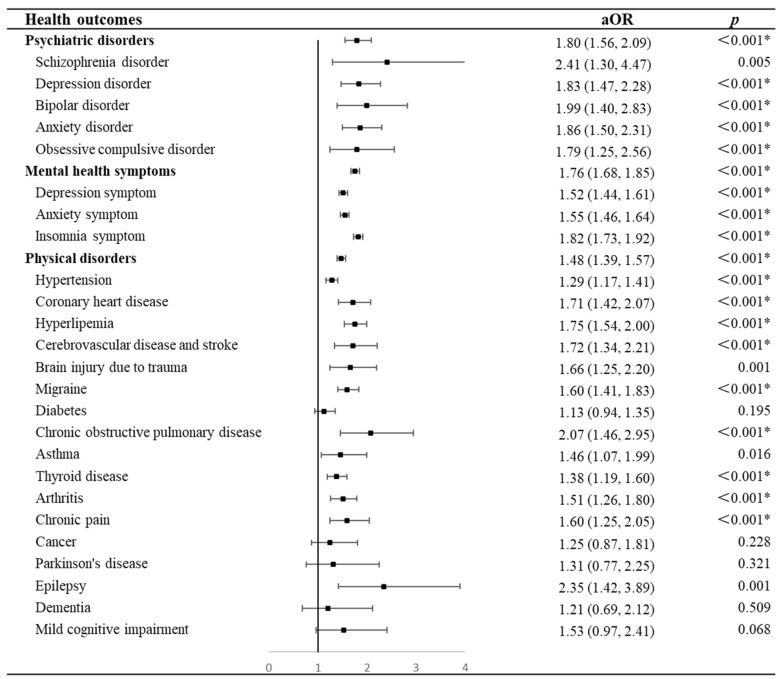
Forest plots of results for odds ratios of health outcomes in shift workers compared with non-shift workers. * *p* < 0.0018 in Bonferroni correction tests for multiple comparisons. Abbreviations: aOR, adjusted odds ratio.

Figure 2 presents the association between shift work and health outcomes in the general population. Compared to non-shift workers, shift workers were more likely to have psychiatric disorders (OR = 1.80, 95% CI = 1.56–2.09, *p* < 0.001), mental health symptoms (OR = 1.76, 95% CI = 1.68–1.85, *p* < 0.001), or physical disorders (OR = 1.48, 95% CI = 1.39–1.57, *p* < 0.001) after adjusting covariates including nocturnal sleep characteristics, day napping, and insomnia, e.g., sleep disturbances. Specifically, shift work exhibited higher ORs of psychiatric disorders, including schizophrenia (OR = 2.41, 95% CI = 1.30–4.47, *p* = 0.005), bipolar disorder (OR = 1.99, 95% CI = 1.40–2.83, *p* < 0.001), anxiety (OR = 1.86, 95% CI = 1.50–2.31, *p* < 0.001), depression (OR = 1.83, 95% CI = 1.47–2.28, *p* < 0.001), and obsessive compulsive disorder (OR = 1.79, 95% CI = 1.25–2.56, *p* = 0.001). Similarly, shift workers were associated with increased ORs of depression symptoms (OR = 1.52, 95% CI = 1.44–1.61, *p* < 0.001), anxiety symptoms (OR = 1.55, 95% CI = 1.46–1.64, *p* < 0.001), and insomnia symptoms (OR = 1.82, 95% CI = 1.73–1.92, *p* < 0.001) when compared with non-shift workers. In addition, we have examined the collinearity and the VIF indicated that no collinearity existed. The detailed results of the VIF are shown in the Supplementary Materials (Table S2).

In addition, shift work was found to raise the likelihood of certain physical disorders, including hypertension (OR = 1.29, 95% CI = 1.17–1.41, *p* < 0.001), coronary heart disease (OR = 1.71, 95% CI = 1.42–2.07, *p* < 0.001), hyperlipidemia (OR = 1.75, 95% CI = 1.54–2.00, *p* < 0.001), cerebrovascular disease or stroke (OR = 1.72, 95% CI = 1.34–2.21, *p* < 0.001), brain injury due to trauma (OR = 1.66, 95% CI = 1.25–2.20, *p* < 0.001), migraine (OR = 1.60, 95% CI = 1.41–1.83, *p* < 0.001), chronic obstructive pulmonary disease (OR = 2.07, 95% CI = 1.46–2.95, *p* < 0.001), asthma (OR = 1.46, 95% CI = 1.07–1.99, *p* < 0.001), thyroid disease (OR = 1.38, 95% CI = 1.19–1.60, *p* < 0.001), arthritis (OR = 1.51, 95% CI = 1.26–1.80, *p* < 0.001), chronic pain (OR = 1.60, 95% CI = 1.25–2.05, *p* < 0.001), and epilepsy (OR = 2.35, 95% CI = 1.42–3.89, *p* < 0.001), but it was not significantly associated with the risk of diabetes (OR = 1.13, 95% CI = 0.94–1.35, *p* = 0.195), cancer (OR = 1.25, 95% CI = 0.87–1.81, *p* = 0.228), Parkinson’s disease (OR = 1.31, 95% CI = 0.77–2.25, *p* = 0.321), dementia (OR = 1.21, 95% CI = 0.69–2.12, *p* = 0.509), and mild cognitive impairment (OR = 1.53, 95% CI = 0.97–2.41, *p* = 0.068).

### 3.3. Association of Frequency of Shift Work with Health Outcomes

**Figure 3:**
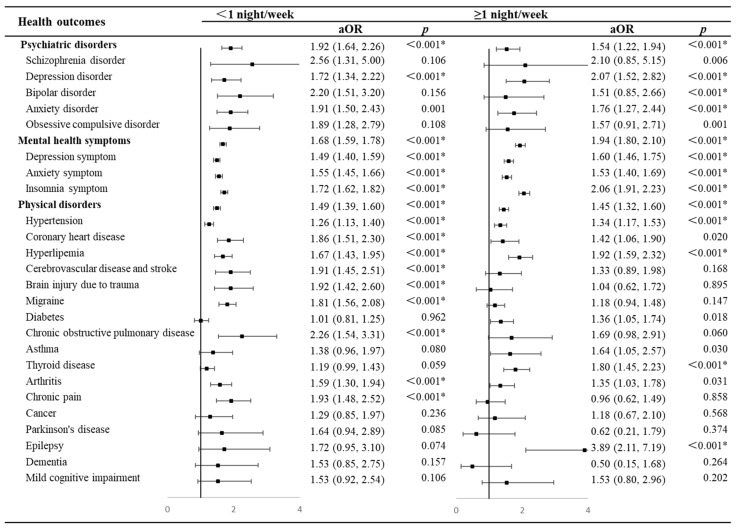
Forest plots of results of odds ratios for health outcomes in different frequencies of shift work compared with non-shift workers. * *p* < 0.0018 in Bonferroni correction tests for multiple comparisons. Abbreviations: aOR, adjusted odds ratio.

Figure 3 demonstrates the association between the frequency of shift work and each health outcome. Compared to non-shift workers, shift workers were associated with an increased likelihood of poor health outcomes regardless of shift frequency. Individuals with a shift frequency of <1 night/week had higher ORs for psychiatric disorders (OR = 1.92, 95% CI = 1.64–2.26, *p* < 0.001 vs. OR = 1.54, 95% CI = 1.22–1.94, *p* < 0.001) and physical disorders (OR = 1.49, 95% CI = 1.39–1.60, *p* < 0.001 vs. OR = 1.45, 95% CI = 1.32–1.60, *p* < 0.001) but lower ORs for mental health symptoms (OR = 1.68, 95% CI = 1.59–1.78, *p* <0.001 vs. OR = 1.94, 95% CI = 1.80–2.10, *p* <0.001) than those with a shift frequency of ≥1 night/week.

### 3.4. Association of Years of Shift Work with Health Outcomes

**Figure 4:**
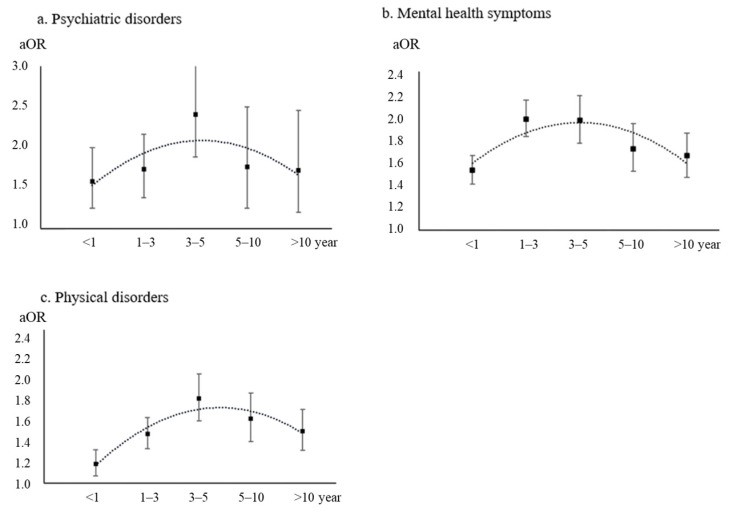
Associations between years of shift work and health outcomes. Abbreviations: aOR, adjusted odds ratio.

Figure 4 presents the results of associations between years of shift work and any health outcomes. Overall, inverted U-shaped associations between years of shift work and health outcomes were observed after adjusting for covariates. The ORs of poor health outcomes associated with shift work rose within 3 years, reached peaks at 3–5 years (psychiatric disorders: OR = 2.41, 95% CI = 1.88–3.10, *p* < 0.001; physical disorders: OR = 1.83, 95% CI = 1.61–2.07, *p* < 0.001), and then declined after more than 5 years of shift work. A similar trend was found for mental health symptoms, with a peak OR at 1–3 years (OR = 1.98, 95% CI = 1.83–2.15, *p* < 0.001), after which the OR began to decline.

## 4. Discussion

This cross-sectional study based on a large national sample focused on the health outcomes of shift workers in China, including mental health status and physical health condition. A total of 23.4% of the respondents had been engaging in shift work in this study. Shift workers were more associated with potential links to poor health outcomes than non-shift workers, neglecting shift frequency. In addition, inverted U-shaped associations were observed between years of shift work and health outcomes. This study provides insight into enhancing the health of shift workers by adjusting their work schedules.

There is a growing interest in the study of shift workers’ status and their health outcomes. A meta-analysis that evaluated seven studies with a total of 28,431 participants pointed out that shift workers were at an increased risk of poor mental health, particularly depression symptoms [13]. In addition, shift work, especially night shift work, was shown to be associated with a higher risk of chronic physical disorders, including diabetes, cardiovascular disease, and cancer [1,40]. Consistent with the findings of previous studies [41,42,43], current results also suggested that shift work was significantly associated with an increased risk of poor health outcomes among the general population in China.

Current research on the mechanisms by which shift work affects health is divided, including the interruption of the normal sleep–wake circadian cycle [44] and shift work tolerance [45,46]. In general speaking, shift work alters the circadian rhythm, causing significant adverse effects on biological functions [5,47]. Moreover, individual differences in tolerance to shift work may also cause distinct results of health outcomes [48]. Individual differences and baseline needs, especially neuroticism and autonomy, have been found to be strongly relevant to the tolerance of shift work in different occupations [49,50]. Irregular work reduces an individual’s ability to adapt to changes in the environment [51], these may be the mechanisms that lead to the link between shift work and chronic disease [22,52,53]. A fuller understanding of how shift work affects physical health remains to be explored.

In this study, we also explored the association between the frequency of shift work and health outcomes. Our results suggested that shift work participation, even with low frequency, may be associated with higher ORs of poor health outcomes. Previous studies have also found that shift work increased the risk of anxiety, depression, and coronary heart disease, neglecting shift frequency [5,54]. However, some studies have found that poor health outcomes increased following more frequent shift work [53]. Notably, we found that some health outcomes did not show a consistent linear relationship with shift frequency, such as cerebrovascular disease, chronic pain, and Parkinson’s disease, which had higher odds with lower shift frequency compared to more frequent shift work. Sleep, circadian rhythms, individual personality, neuroticism, and autonomy are all potential contributing factors to this outcome [49]. In addition, variations in study design, sample size, and work intensity may also be responsible [5,53,55]. In the meantime, it should be pointed out that the frequency of shift work in this study was not concise (merely divided into <1 and ≥1 night/week), and a precise estimate of the average number of shifts per month was needed.

When exploring the relationship between years of shift work and health outcomes, U-shaped associations were observed. Similarly, one study found that the relationship between shift work and cardiovascular disease risk appeared to emerge only after the first 5 years of exposure to shift work, after which it would gradually decrease [41]. Meanwhile, a study on the healthy age of female nurses in the U.S. also found that 1–5 years of shift work was a risk factor for healthy aging [21]. Work tolerance may be an explanation. The research has shown that shift work can lead to fatigue at the start of a shift, but an increased work capacity can help reduce fatigue and promote recovery from poor health outcomes [56]. Although the ORs for poor health outcomes decreased after five or more years, the risks still remained, suggesting that the structure of shift work needs to be improved to mitigate these hazards.

Our study has some strengths. To our knowledge, this cross-sectional study with a large sample of the general Chinese population provided an opportunity to observe the association between shift work and health outcomes more comprehensively. Our results suggested that there was no consistent linear relationship between shift frequency and health outcomes, and inverted U-shaped associations were observed between years of shift work and health outcomes. Our findings may provide more helpful information for policy making of shift work and recognition of high-risk disease progression in shift workers. Nonetheless, the study has several limitations as well. First, this was an online survey, and we used a convenience sampling method. Although the questionnaire was collected nationally, internet users are mostly highly educated young people, and the representativeness of our sample may be limited. Second, some other aspects of shift work such as the relationship with rotation work, overtime on holidays, frequency, pattern, or work type were not well detected in the current study. Third, this study was cross-sectional; hence, we were not able to explore the causal relationship between the frequency of shift work and adverse health outcomes. Fourth, the health outcomes we collected, including mental health and physical health, were derived from respondents’ self-reports rather than clinical diagnoses, which are subject to recall bias and may lead to error. And we investigated only common physical and mental illnesses, and a more detailed history of the disease was not explored among our participants.

## 5. Conclusions

Shift work is associated with a higher likelihood of poor health outcomes in the general population in China. The current research findings emphasize the necessity of paying attention to the health status of shift workers and it is encouraged to take effective measures to mitigate the negative impact of shift work and promote public health. The frequency, intensity, and duration of shift work, as well as the type of shift, need to be further considered in the following cohort studies in order to obtain a full picture of shift work.

## Figures and Tables

**Table 1:** Demographic characteristics of the total sample stratified by shift work.

	Non-Shift Workers (N = 31,449)	Shift Workers (N = 9612)	Total Sample (N = 41,061)	*p*
Age (mean ± SD)	36.59 ± 9.73	35.09 ± 8.66	36.24 ± 9.51	<0.001 *
Sex				<0.001 *
Male	11,023 (35.1)	5791 (60.2)	16,814 (40.9)	
Female	20,426 (64.9)	3821 (39.8)	24,247 (59.1)	
Ethnicity				0.013
Ethnic Han	30,426 (96.7)	9249 (96.2)	39,675 (96.6)	
Ethnic minorities	1023 (3.3)	9363 (3.8)	1386 (3.4)	
Living area				<0.001 *
Rural	2456 (7.8)	942 (9.8)	3398 (8.3)	
Urban	28,993 (92.2)	8670 (90.2)	37,663 (91.7)	
Education attainment				<0.001 *
High school or lower	5663 (18.0)	2121 (22.1)	7784 (19.0)	
University or college degree (%)	23,265 (74.0)	5043 (68.7)	29,874 (72.8)	
Postgraduate	2521 (8.0)	882 (9.2)	3403 (8.3)	
Marital status				<0.001 *
Unmarried	6970 (22.2)	2609 (27.2)	9579 (23.3)	
Married	23,748 (75.5)	6766 (70.4)	30,514 (74.3)	
Separate, widow, or others	723 (2.3)	234 (2.4)	968 (2.4)	
Type of jobs ^a^				<0.001 *
Non-manual work	20,692 (65.8)	6328 (65.8)	27,020 (65.8)	
Manual work	5234 (16.6)	2160 (22.5)	7394 (18.0)	
Jobless or retired	5523 (17.6)	1124 (11.7)	6647 (16.2)	
Income level (CNY/month)				<0.001 *
≤4999	9621 (30.6)	2658 (27.7)	12,279 (29.9)	
5000–19,999	16,631 (52.9)	5453 (56.7)	22,084 (53.8)	
≥20,000	5197 (16.5)	1501 (15.6)	6698 (16.3)	
Smoking				<0.001 *
Yes	3073 (9.8)	2035 (21.2)	3738 (9.1)	
No	28,376 (90.2)	7577 (78.8)	37,323 (90.9)	
Drinking				<0.001 *
Yes	1628 (5.2)	1012 (10.5)	2640 (6.4)	
No	29,821 (94.8)	8600 (89.5)	38,421 (93.6)	
Diet ^b^				<0.001 *
Healthy	15,794 (50.2)	3970 (41.3)	19,764 (48.1)	
Unhealthy	15,655 (49.8)	5642 (58.7)	21,297 (51.9)	
BMI				<0.001 *
Normal	17,771 (56.5)	4819 (50.1)	22,590 (55.0)	
Abnormal	13,678 (43.5)	4793 (49.9)	18,471 (45.0)	
Exercise habit ^c^				<0.001 *
Yes	8114 (25.8)	2280 (23.7)	10,394 (25.3)	
No	23,335 (74.2)	7332 (76.3)	30,667 (74.7)	
Sedentary behavior ^d^				<0.001 *
High	20,401 (64.9)	5724 (59.6)	26,125 (63.6)	
Low	11,048 (35.1)	3888 (40.4)	14,936 (36.4)	
Napping habit ^e^				<0.001 *
No	6290 (20.0)	1828 (19.0)	8118 (19.8)	
Less frequently	10,297 (32.7)	3958 (41.2)	14,255 (34.7)	
Frequently	14,862 (47.3)	3826 (39.8)	18,688 (45.5)	
Have you ever been infected with COVID-19?				<0.001 *
No	30,476 (96.9)	9100 (94.7)	39,576 (96.4)	
Asymptomatic infection	483 (1.5)	324 (3.4)	807 (2.0)	
Confirmed infection	490 (1.6)	188 (1.9)	678 (1.7)	
Have you ever been quarantined during COVID-19 epidemics?				<0.001 *
No	29,331 (93.3)	8705 (90.6)	38,036 (92.6)	
Ever quarantined	1852 (5.9)	789 (8.2)	2641 (6.4)	
Being quarantined currently	266 (0.8)	118 (1.2)	384 (0.9)	

Abbreviations: BMI: body mass index; COVID-19: coronavirus disease 2019. ^a^: non-manual work included civil servants, institutional staff, enterprise or company employees, freelancers (e.g., writers, artists, anchors, etc.), medical workers; manual work included private or individual workers, service workers, servicemen or military occupations, workers, and farmers; jobless or retired included students, full-time caretaker of a family, unemployed or jobless, and retired; ^b^: a healthy diet was defined as frequently taking at least four of seven healthy foods (≥4 days a week) and seldom taking above two unhealthy foods (≤3 days a week), otherwise was defined as unhealthy diet; ^c^: for physical exercise, at least 150 min of mild or 75 min of moderate-to-vigorous activity per week was considered healthy, otherwise was defined as unhealthy; ^d^: sedentary behavior ≤ 6 h a day was defined as low, equal or >6 h was defined as high; ^e^: napping occasionally (1–3 times/month) and sometimes (1–2 times/week) were defined as less frequent napping habit; napping often (3–5 times/week) and almost every day were defined as frequent napping habit. * *p* < 0.0029 in Bonferroni correction tests for multiple comparisons.

## Data Availability

The data and statistical analysis plan in this study are available to others after publication. To access this data, please email one of the corresponding authors.

## Supplementary Materials

The following supporting information can be downloaded at https://www.mdpi.com/article/10.3390/brainsci14020145/s1: Table S1 Proportions of health outcomes and X^2^ in shift workers and non-shift workers. Table S2: The variance inflation factors of variables in the model.
