# Supplementary material for: Association between Shift Work and Health Outcomes in the General Population in China: A Cross-Sectional Study
Source: Brain Sci. 2024 Jan 30;14(2):145. doi: 10.3390/brainsci14020145 (PMC10886504; doi:10.3390/brainsci14020145)
Supplement: Supplementary file 1 [file brainsci-14-00145-s001.zip › brainsci-2791890-supplementary.pdf]

Association between shift work and health outcomes in the general population in China: a cross-sectional study

Nan Gao [1,2,#], Yong-Bo Zheng, [2,3,#] Ying-Bo Yang [1], Yue-Tong Huang, [2,3] San-Wang Wang [2,4] and Na Zeng, [5,6] Shu-Yu Ni, [5,6] Shui-Lin Wu, [5,6] Si-Zhen Su, [2] Zhi-Bo Zhang, [2] Kai Yuan, [2] Le Shi, [2] Zhao-Hui Zhang, [1,*] Wei Yan, [2,*] Lin Lu, [2,3,5,*] Yan-Ping Bao [5,6,*]

Table S1 Proportions of health outcomes and X^2 in shift workers and non-shift workers.

	Non-shift work	Shift work	F	P-value
Psychiatric disorders	528(1.68%)	355(3.69%)	137.233	<0.001*
Depression disorder	243(0.77%)	145(1.51%)	42.589	<0.001*
Bipolar affective disorder	75(0.24%)	72(0.75%)	53.801	<0.001*
Anxiety disorder	238(0.76%)	158(1.64%)	60.643	<0.001*
Obsessive-compulsive disorder	80(0.25%)	62(0.65%)	32.599	<0.001*
Schizophrenia	23(0.07%)	24(0.25%)	20.071	<0.001*
Mental health symptoms	11692(37.18%)	5017(52.20%)	687.957	<0.001*
Insomnia symptom	8935(28.41%)	4048(42.11%)	639.347	<0.001*
Depression symptom	5447(17.32%)	2512(26.10%)	365.993	<0.001*
Anxiety symptom	4616(14.68%)	2251(23.40%)	403.875	<0.001*
Physical disorders	5009(15.93%)	2105(21.90%)	183.326	<0.001*
Hypertension	2097(6.67%)	883(9.19%)	69.376	<0.001*
Hyperlipemia	749(2.38%)	409(4.26%)	94.282	<0.001*
Migraine	828(2.63%)	382(3.97%)	46.315	<0.001*
Thyroid disease	889(2.83%)	252(2.62%)	1.146	0.284
Coronary heart disease	329(1.05%)	214(2.23%)	78.586	<0.001*
Arthritis	479(1.52%)	202(2.10%)	15.103	<0.001*
Diabetes	505(1.61%)	187(1.95%)	5.128	0.024
Cerebrovascular disease and stroke	172(0.55%)	122(1.27%)	54.034	<0.001*
Chronic pain	194(0.62%)	112(1.17%)	29.926	<0.001*
Brain injury due to trauma	126(0.40%)	95(0.99%)	47.499	<0.001*
Asthma	148(0.47%)	66(0.69%)	6.627	0.010
Chronic obstructive pulmonary disease	79(0.25%)	64(0.67%)	36.469	<0.001*
Cancer	135(0.43%)	43(0.45%)	0.056	0.813
Epilepsy	33(0.10%)	38(0.40%)	35.969	<0.001*
Mild cognitive impairment	53(0.17%)	36(0.37%)	14.445	<0.001*
Parkinson's disease	37(0.12%)	26(0.27%)	11.227	<0.001*
Dementia	36(0.11%)	23(0.24%)	7.993	0.005

*p <0.0018 in Bonferroni correction tests for multiple comparison.

Table S2: The variance inflation factors of variables in the model

	VIF
Age	1.432
Sex	1.292
Ethnicity	1.002
Living area	1.066
Education attainment	
University or college degree	1.495
Postgraduate	1.524
Marital status	
Married	1.169
Separate, widow, or others	1.480
Type of jobs	
Manual work	1.144
Jobless or retired	1.265
Income level (yuan/month)	
5000-19999	1.576
≥20000	1.468
Smoking	1.303
Drinking	1.140
Diet	1.049
BMI	1.022
Exercise habit	1.057
Sedentary behavior	1.830
Napping habit	1.811
Have you ever been infected with COVID-19?	
Asymptomatic infection	1.041
Confirmed infection	1.216
Have you ever been quarantined during COVID-19 epidemics?	
Ever quarantined	1.226
Being quarantined currently	1.030
